# Editorial: Male fertility: lessons learnt from the SARS-CoV-2 pandemic

**DOI:** 10.3389/fphys.2024.1372217

**Published:** 2024-02-26

**Authors:** Stefan S. Du Plessis, Eva Tvrdá, Walter D. Cardona Maya, Temidayo S. Omolaoye

**Affiliations:** ^1^ Department of Basic Sciences, College of Medicine, Mohammed Bin Rashid University of Medicine and Health Sciences, Dubai, United Arab Emirates; ^2^ Institute of Biotechnology, Faculty of Biotechnology and Food Sciences, Slovak University of Agriculture in Nitra, Nitra, Slovakia; ^3^ Reproduction Group, Department of Microbiology and Parasitology, Faculty of Medicine, University of Antioquia-UdeA, Medellín, Colombia

**Keywords:** SARS-CoV-2, male fertility, male reproductive health, male sexual health, hormones, proteomics, reproductive system

The severe acute respiratory syndrome coronavirus 2 (SARS-CoV-2), which caused the COVID-19 pandemic left a lasting effect on global health, prompting researchers to delve into the multifaceted repercussions of the virus on various physiological systems. During the initial discovery phase, SARS-CoV-2 was reported to be detected in saliva, respiratory fluids, blood, urine, and faeces (Mohseni et al., 2020), necessitating investigations on transmission through these routes. This then begged the question whether SARS-CoV-2 is present in the semen, and if present, would the viral shedding have transmission potency? The concern of SARS-CoV-2 transmission through the semen emanated from how previously identified viruses such as Ebola and Zika virus were sexually transmitted (Cardona Maya et al., 2016; McCarthy, 2016; Deen et al., 2017; Cardona-Maya et al., 2019). However, findings on this matter soon became controversial, as an initial study from Li et al. reported the presence of SARS-CoV-2 in the semen samples of acutely infected or recovered patients (Li et al., 2020). It was furthermore shown that SARS-CoV-2 was present in the *postmortem* testes of patients with COVID-19 (Yang et al., 2020). Subsequent findings however reported the absence of SARS-CoV-2 in the semen of infected men (Guo et al., 2020; Kayaaslan et al., 2020; Pan et al., 2020). Despite the controversy regarding its presence in the semen, it was evident that men infected with SARS-CoV-2 displayed abnormal semen parameters, altered hormone profiles, and disrupted spermatogenesis. These adverse effects are highlighted and explained in detail in earlier review articles (Omolaoye et al., 2021; Omolaoye et al., 2022).

Since the virus has been shown to impose adverse effects on both the structural components and function of the testis, which potentially impact male fertility, the aim of this Research Topic of manuscripts was to provide recent updates on the status of SARS-CoV-2 on male fertility, by exploring the different pathophysiological and sub-cellular mechanisms that can impact reproductive potential.

To this effect, manuscripts included in this Research Topic provides intriguing insights into the interplay between COVID-19 and male fertility, offering lessons that extend beyond the immediate concerns of the pandemic.

In their minireview Ly et al. explored the impact of SARS-CoV-2 beyond its well-documented respiratory effects. They present findings from both autopsy cases and animal models that investigated testicular disease associated with COVID-19. Notably, the male reproductive tract has shown histopathological lesions and disruptions of the blood-testes barrier.

Building on the minireview, Alzahrani et al. examined studies that investigated the repercussions of the COVID-19 pandemic on men’s sexual and reproductive health. Through an in-depth literature review, the potential pathophysiological impact of SARS-CoV-2 on testicular, epididymal, prostatic, and penile tissues were evaluated. Following this comprehensive search, findings showed the adverse consequences of SARS-CoV-2 at cellular and organ levels within the male reproductive tract, thus ascertaining the virus’ negative effect on male fertility potential. Additionally, they noted that men infected with SARS-CoV-2 experience more severe illness and higher fatality rates than women. Hence, understanding the gender-based susceptibility to severe diseases can inform the development of effective therapies, public health policies, and targeted strategies, such as early detection and intensive testing in specific subgroups.

Schmidt et al. explored the clinical manifestations of COVID-19, particularly focusing on the exacerbated immune response, cytokine storm, and poorly understood prodromal effects of autoantibodies. They screened two independent cohorts of COVID-19 patients (Discovery cohort from Qatar and Replication cohort from New York). The study identified significantly altered autoantibody responses to proteins in the COVID-19 cohorts when compared to respective controls. Both cohorts exhibited substantial similarities and elevated autoantibody responses to transcription factors, immunomodulatory proteins, and human disease markers. Combined analysis revealed increased autoantibody responses against specific proteins (SPANXN4, STK25, ATF4, PRKD2, and CHMP3) in COVID-19 patients. As SPANXN4 is crucial for spermiogenesis and male fertility, their study suggests a potential role for this protein in COVID-19-associated male reproductive tract complications, emphasizing the need for further research.

Complementing the auto-immunoproteomics analysis, a manuscript by Dash et al. delves into the aftermath of the infection on male fertility. They investigated the impact of SARS-CoV-2 and its variants on male reproductive health, focusing on changes in sperm quality and reproductive function in recovered individuals. Proteomic analysis revealed dysregulation of 69 proteins, accompanied by altered semen parameters in COVID-19-recovered patients. Pathways related to exocytosis, neutrophil degranulation, immune response, spermatogenesis, and calcium ion transport, which directly or indirectly affect sperm parameters were also dysregulated. The study suggests that the inflammatory flare-up witnessed during active COVID-19 infection persists even after clinical recovery. This persistence underscores the notion that post-COVID-19 sequelae are influenced by lingering inflammatory responses, irrespective of the prevalent virus variants or vaccination status. Thus, more in-depth research is needed to address long-term consequences transcending beyond immediate recovery.

Additionally, Martinez et al. prospectively investigated the impact of COVID-19 on the male reproductive system. Examining 231 reproductive-aged male patients who had recovered from mild or severe COVID-19, along with 62 healthy controls, the study assessed sperm quality parameters in addition to inflammatory cytokines, and leukocytes in semen. Unlike many reported studies, this research benefits from extensive baseline data for a more accurate analysis of COVID-19 effects on sperm quality. Findings reveal significantly elevated levels of inflammatory cytokines (IL-1β, TNF, and IFNγ) in semen from recently recovered patients, indicating semen inflammation. Moreover, patients displayed altered semen parameters. Importantly, these alterations returned to baseline values after 3 months of post-disease recovery, suggesting reversible effects. Findings from this study offer vital information on the sequelae associated with COVID-19, particularly relevant to Research Topic of semen for use during assisted reproduction.

Collectively, these studies illuminate the intricate relationship between COVID-19 and male fertility, prompting reflections on the lessons learnt during the pandemic. The manuscripts discussed herein not only contribute to our understanding of COVID-19’s impact on male fertility but also emphasize the necessity for ongoing research, multidisciplinary collaboration, and a nuanced approach to address the complexities of reproductive health during and after the pandemic. As we navigate the challenges posed by SARS-CoV-2, the lessons learnt from these studies can guide future research, public health initiatives, and clinical care, ensuring a comprehensive approach to male reproductive health in the post-COVID era.
